# High-Protein Diet in Lactation Leads to a Sudden Infant Death-Like Syndrome in Mice

**DOI:** 10.1371/journal.pone.0017443

**Published:** 2011-03-09

**Authors:** Thomas Walther, Nils Dietrich, Martina Langhammer, Marzena Kucia, Harald Hammon, Ulla Renne, Wolf-Eberhard Siems, Cornelia C. Metges

**Affiliations:** 1 Department Experimental Cardiology, Excellence Cluster Cardio-Pulmonary System, Justus-Liebig-Universität Giessen, Giessen, Germany; 2 Centre for Biomedical Research, Hull York Medical School, University of Hull, Hull, United Kingdom; 3 Research Unit Nutritional Physiology, Leibniz Institute for Farm Animal Biology (FBN), Dummerstorf, Germany; 4 Freie Universität Berlin, Berlin, Germany; 5 Leibniz-Institut für Molekulare Pharmakologie (FMP), Berlin, Germany; Sapienza University of Rome, Italy

## Abstract

**Background:**

It is well accepted that reduced foetal growth and development resulting from maternal malnutrition are associated with a number of chronic conditions in later life. On the other hand such generation-transcending effects of over-nutrition and of high-protein consumption in pregnancy and lactation, a proven fact in all developed societies, are widely unknown. Thus, we intended to describe the generation-transcending effects of a high-protein diet, covering most relevant topics of human life like embryonic mortality, infant death, and physical health in later life.

**Methods:**

Female mice received control food (21% protein) or were fed a high protein diet (42% protein) during mating. After fertilisation, females stayed on their respective diet until weaning. At birth, pups were put to foster mothers who were fed with standard food or with HP diet. After weaning, control diet was fed to all mice. All offspring were monitored up to 360 days after birth. We determined glucose-tolerance and measured cardiovascular parameters using a tip-catheter. Finally, abdominal fat amount was measured.

**Results and Conclusions:**

We identified a worried impact of high-protein diet during pregnancy on dams' body weight gain, body weight of newborns, number of offspring, and also survival in later life. Even more important is the discovery that high-protein diet during lactation caused a more than eight-fold increase in offspring mortality. The observed higher newborn mortality during lactation is a hitherto non-described, unique link to the still incompletely understood human sudden infant death syndrome (SIDS). Thus, although offspring of lactating mothers on high-protein diet might have the advantage of lower abdominal fat within the second half of life, this benefit seems not to compensate the immense risk of an early sudden death during lactation. Our data **may** implicate that both pregnant women and lactating mothers should not follow classical high-protein diets.

## Introduction

“Barker Hypothesis” states that reduced foetal growth and development resulting from maternal malnutrition are associated with a number of chronic conditions in the later life of mammals [1]. On the other hand, modern life in developed societies is not characterized by malnourishment, but conversely by overnutrition, associated with the development of an obese phenotype. It has been postulated that this should also result in health disturbance in the next generation. Observational human studies seem to confirm such postulates; but exact verifiable acquisitions of human data require decades and enormous work to reach statistically robust conclusions [2], [3], [4]. Consequently, customized animal studies with mice or rats may clarify such postulated connections. Very recently, Chang *et al.* [5] described that maternal high-fat diet in rats results in long-term behavioural and physiological changes in offspring including increase of food intake, preference for fat, hyperlipidemia, and higher body weight. The mouse study presented here was initiated to determine the short- and long-term effects of a maternal high-protein diet in pregnancy or lactation on offspring's body weight, fat accumulation and cardiovascular parameters after weaning.

As a result of our study we found the predicted, significant influence of the maternal nutrition on the development of offspring in their later life. However, much more dramatic are direct effects initiated by nutrition of pregnant animals reducing the number of live-born pups, and by the nutrition of dams during lactation significantly increasing the mortality of sucklings. From the latter we conclude that a high-protein diet during lactation strongly promotes the incidence of a sudden infant death-like syndrome. These findings point to a fundamentally new approach for the research on and prevention of redoubtable sudden infant death syndrome (SIDS) [6], [7], [8].

## Materials and Methods

### Mice line

Outbred line DUK [9], [10] was bred at the Leibniz Institute for Farm Animal Biology (FBN) in Dummerstorf, Germany.

Experiments on adult mice were performed in accordance with the Guide for the Care and Use of Laboratory Animals published by the US National Institutes of Health (NIH Publication No. 85-23, revised 1996) and the Federal Law on the Use of Experimental Animals in Germany, and were approved by the local authorities (Reg.-Nr. LALLF M-V/TSD/7221.3-1.1-033/06; Reg.-Nr. G 0258/05).

### Sets of experimental mouse groups

**Figure 1:**
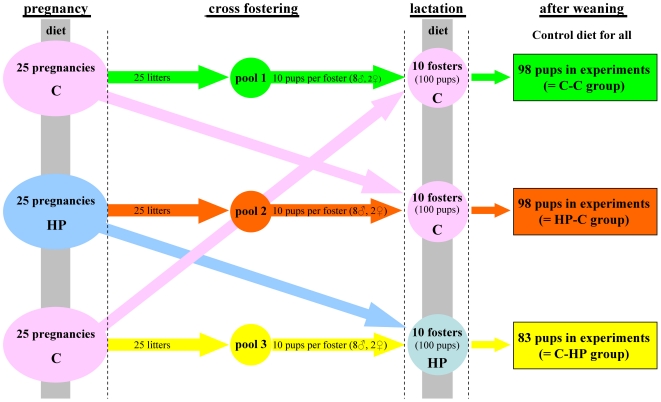
Scheme describing the generation of the different diet groups. Detailed description on the generation of the C-C, C-HP, and HP-C groups has been given in the Materials and Methods and the Results parts. C  =  control diet, HP  =  high-protein diet.

Fifty male and female mice received control food (C, 21% protein) and 25 males and females were fed a high protein diet (HP, 42% protein) during mating (**Figure 1**). Mice were allocated to the two experimental diets in a random manner; experimental diets were fed already during conception. Males were withdrawn immediately after confirmation of pregnancy (appearance of a vaginal plug), which was denoted day 1 of pregnancy. After fertilisation/impregnation, females stayed on their respective diet until weaning. At birth, pups were allocated to standardized litters (8♂, 2♀) and put back to foster mothers (with delivery, mothers became foster mothers and were scaled down to 20 with C- and 10 with HP-diet). Consequently, the resulting experimental groups have been as followed:

- Offspring born to mouse dams fed control diet (C) during pregnancy was cross-fostered by dams that received the high protein (HP) diet during pregnancy and continued on the HP diet during lactation (C-HP),
- A group whose mothers were given the HP diet during pregnancy was cross-fostered by dams fed control diet throughout pregnancy and lactation (HP-C), and
- A control group (C-C) with mothers fed control diet in pregnancy was cross-fostered by dams fed control diet in pregnancy and lactation.

Pregnant and lactating dams were housed individually in standard rodent cages (Makrolon, Type II, EBECO, Castrop-Rauxel, Germany) in a controlled environment of 22°C with a 12∶12 h light/dark cycle. Water was available *ad libitum*.

### Diets

Two isoenergetic (16.3 MJ metabolizable energy/kg dry matter) semi-synthetic experimental diets with control and high protein level were fed. The two different diets consisted of casein (Molkereigesellschaft Lauingen mbH, Lauingen, Germany; C, 212 g/kg; HP 426 g/kg) supplemented with DL-methionine (4 g/kg; LAH GmbH & CO. KG, Cuxhaven, Germany), wheat starch (Ferdinand Kreutzer Sabamühle GmbH, Nürnberg, Germany; C, 443.9 g/kg; HP 225.9 g/kg), sucrose (Nordzucker GmbH, Hamburg, Germany; 160 g/kg), soya oil (Sedina ADM, Hamburg, Germany; 50 g/kg), microcellulose (50 g/kg), vitamin mixture (20 g/kg; SSNIFF Spezialdiäten GmbH, Soest, Germany), mineral mixture (60 g/kg; SSNIFF Spezialdiäten GmbH) and butylhydroxytoluene (0.1 g/kg; LAH GmbH & CO. KG).

After weaning, a standard rodent diet was fed to all mice (21% protein, 0.4% L-Met, 55% starch, 5% sucrose, 5% fat; 5% cellulose, 2% vitamin and 6% mineral mixture; Altromin 1314, Altromin Spezialfutter GmbH & Co. KG, Lage, Germany).

### Glucose tolerance test

At day 150, mice (n≥7 per group) were starved for 12 h. They were fed a glucose solution (1 g/kg body weight; concentration of glucose solution: 200 mg/ml) via oral gavage. Blood samples were taken from the tail-tip. Blood glucose measurement was performed with a Glucometer (Bayer Ascensia Elite®). Values were taken 30 min before feeding (fasted value) and again 5, 10, 15, 30, 60, 120, 180, 240, 300 and 360 min post glucose challenge.

### Cardiovascular characterization via tip-catheter

At day 360, tip-catheter was inserted via Arteria carotis, Arcus aortae, and Valva aortae into the left ventricle as described previously (n≥7 per group) [11]. Measurement included e.g. heart rate (HR), left ventricular pressure (LVP), contraction, systolic blood pressure (SBP) and diastolic blood pressure (DBP).

### Body weight and abdominal fat determination

Litters were weighted day 1 after birth, and single mice at weaning (day 21), day 180, and day 360. After culling the mice, abdomen was opened up, and fat pad connected to testicles was taken and weighted (n = 8 per group).

### Expression of whey acidic protein expression

Whey acidic protein (WAP) expression has been measured on mRNA level as described earlier (n≥8 per group) [12].

### Statistics

Statistical calculations were performed using Prism or Instat software from GraphPad, San Diego (USA). Comparison of means was determined by student t tests. The variances of results were calculated by ANOVA tests. Significant differences between selected groups were calculated by Bonferroni posttests. To calculate the significances in the survival during lactation, the Friedman test has been used. Kaplan-Meier survival curves were used to compare the survival rate between the groups, and logrank test was performed to analyze the statistical difference. *P*<0.05 values were considered to be statistically significant.

## Results and Discussion

To investigate the relation between maternal diet composition and offspring growth development and health status, we generated, as described in Materials and Methods, three offspring groups depending on the diet of their mother during pregnancy or their foster mother during lactation.

**Figure 2:**
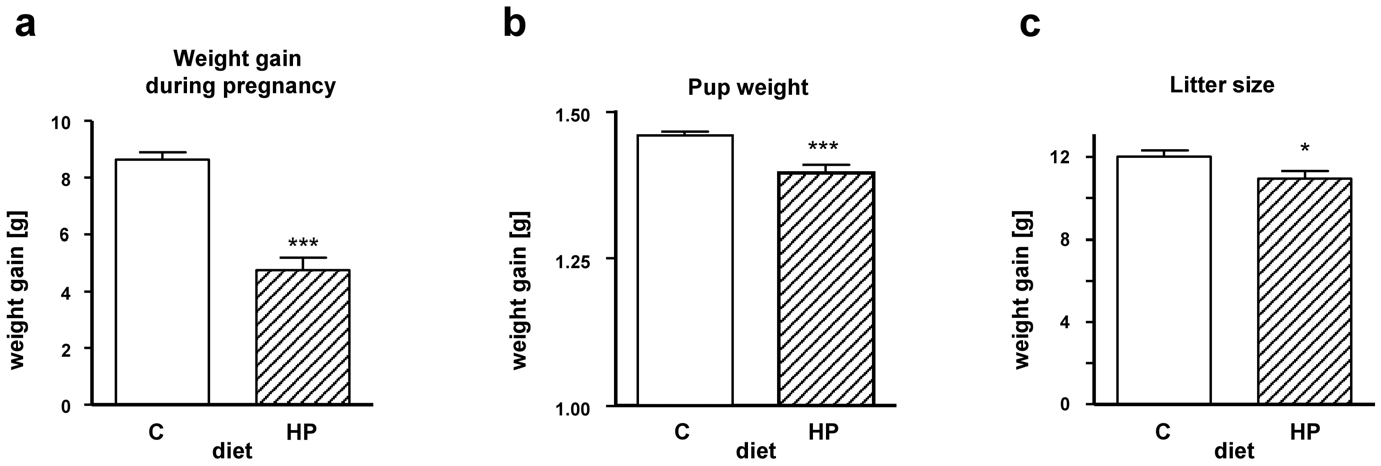
Effect of diets during pregnancy and birth. At day 1 after birth, mothers and litters were examined (group C [n = 50] as the base for C-C and C-HP, group HP [n = 25] is the base for HP-C). (**a**) Body weight gain of females during pregnancy as difference of weight after delivery vs. day of mating; Litter size (**b**) and birth weight (**c**) depending on diet of mother during pregnancy. Data are the means ± SEM, *P<0.05, ***P<0.001 vs control food (C) using a Student's *t*-test.

On initiation of the study, 50 (2×25) females at fertilization were fed with control diet, whereas 25 fertilized mice were on high-protein diet (isoenergetic to control diet; **Figure 1**). The body weight gain of the dams under high-protein diet during pregnancy (weight after delivery minus weight before pregnancy) was only half of that of the dams on a control diet (**Figure 2a**). Importantly, the impact on body weight was seen in both the pregnant animals and also in their offspring. As shown in **Figure 2b**, body weight of the newborns was significantly lower than that of newborns where mothers were on the control diet. Interestingly, the number of pups per litter was also significantly lower in the high-protein fed group (**Figure 2c**), implicating an effect of that diet on intrauterine development and mortality. This is in accord with previously reported studies, showing that higher maternal dietary protein intake in pregnancy is associated with lower ponderal index at birth [13].

Immediately after birth, litters were standardised to 10 pups per dam (8 males, 2 females per litter). Thus, each of the three diet groups carried 80 male and 20 female offspring until weaning (21^st^ day of lactation; **Figure 1**).

**Figure 3:**
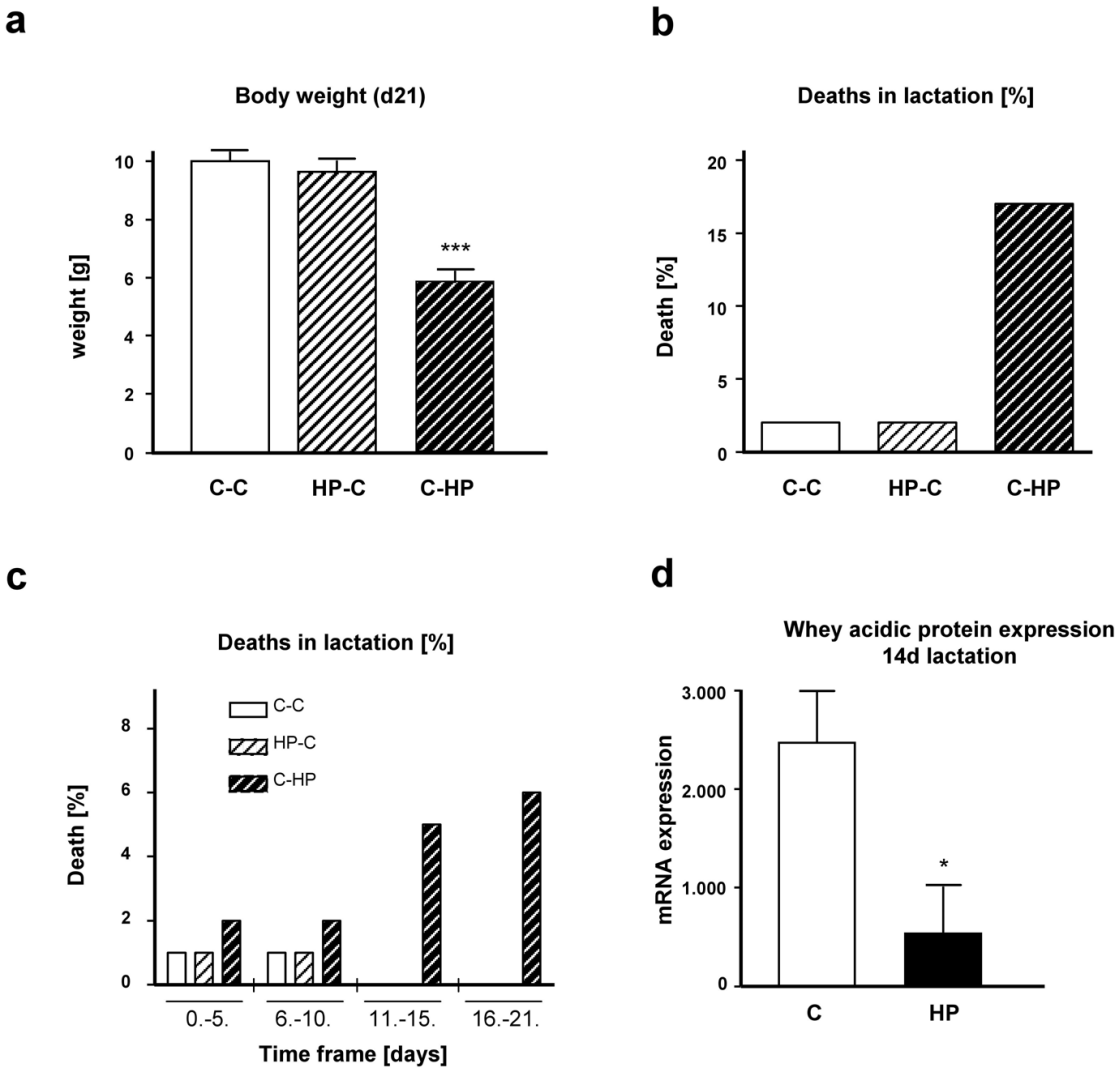
Effect of diets during lactation on body weight, survival and milk composition. Litters were cross-fostered and standardized (8♂, 2♀) at birth. Resulting from dietary treatments 80 males and 20 females counted per group (control-control: C-C, high protein-control: HP-C, and control-high protein C-HP). (**a**) Pup body weight at weaning (21^st^ day of life) depending on diet group (C-C: 98 pups; HP-C: 98 pups; C-HP: 83 pups). (**b**) Number of death in lactation in the three examined groups shown in % (of 100 pups) for period from birth till weaning and (**c**) Number of death in lactation in the same period subdivided in five-day steps; (**d**) mRNA levels of whey acidic protein in mammary glands was measured in lactating mice on control diet (C; n = 8) or high-protein diet (HP; n = 9). Data are the means ± SEM; group differences are calculated by ANOVA (**3a**), Friedman test (**3c**; *P* = 0.018 C-HP *vs* C-C and HP-C) or Student's t-test (**3d**), *P<0.05 *vs* control food (C), ***P<0.001 *vs* control group (C-C).

Although newborns from HP pregnancies were characterized by lower body weight (**Figure 2b**), this difference was abrogated until weaning due to the switch to a foster mother that was fed control diet during pregnancy and lactation (**Figure 3a**), suggesting that HP diet during pregnancy has a less impact on body weight gain at weaning than HP diet during pregnancy. This was further supported by the finding that the newborns from the control diet group during prenatal development, exposed to HP foster mothers only after delivery (C-HP), were characterized by massively lower body weight at weaning in comparison to the group exposed to HP diet during *in utero* life and with fosters, which received control diet during lactation (**Figure 3a**).

The most striking finding in the offspring group with high-protein diet during lactation was a significant association with sucklings' survival (*P* = 0.018). During lactation, 17% of all C-HP pups died (17 pups), whereas only 2% of the pups suckled by control-fed dams died (2 pups, **Figure 3b**), independently of their dietary exposure during *in utero* life. Notably, prior to death, none of the sucklings was characterized by obvious signs of discomfort or illness. This data clearly points to a high risk for pups to die during lactation suckled by dams (foster mothers) fed with high-protein diet. This is further supported by detailed analyses of the occurrence of sucklings' death during the lactation period. Divided in 5-day blocks, difference in survival occurred within the whole lactation phase, with highest difference within the second half (**Figure 3c**) where mortality further increased in the C-HP group while no further mortality has been observed in both other groups. Notably, the higher incidence for death has been distributed over all litters on high-protein diet.

Since investigations on dead sucklings did not reveal morphological changes, we sought for explanations for the sudden death in the C-HP group. Whey acidic protein (WAP) is the major milk protein in certain mammals including mice. In an independent study, we measured mRNA concentration in mammary glands at peak lactation (day 14 of the lactation period) and found only less than 25% of WAP mRNA abundance in foster mothers receiving HP diet throughout pregnancy and lactation compared to mammary glands isolated from mothers on control diet (**Figure 3d**). Thus, our data implicates a clear relation between high-protein diet during pregnancy/lactation, lactogenesis, and sucklings' mortality.

**Figure 4:**
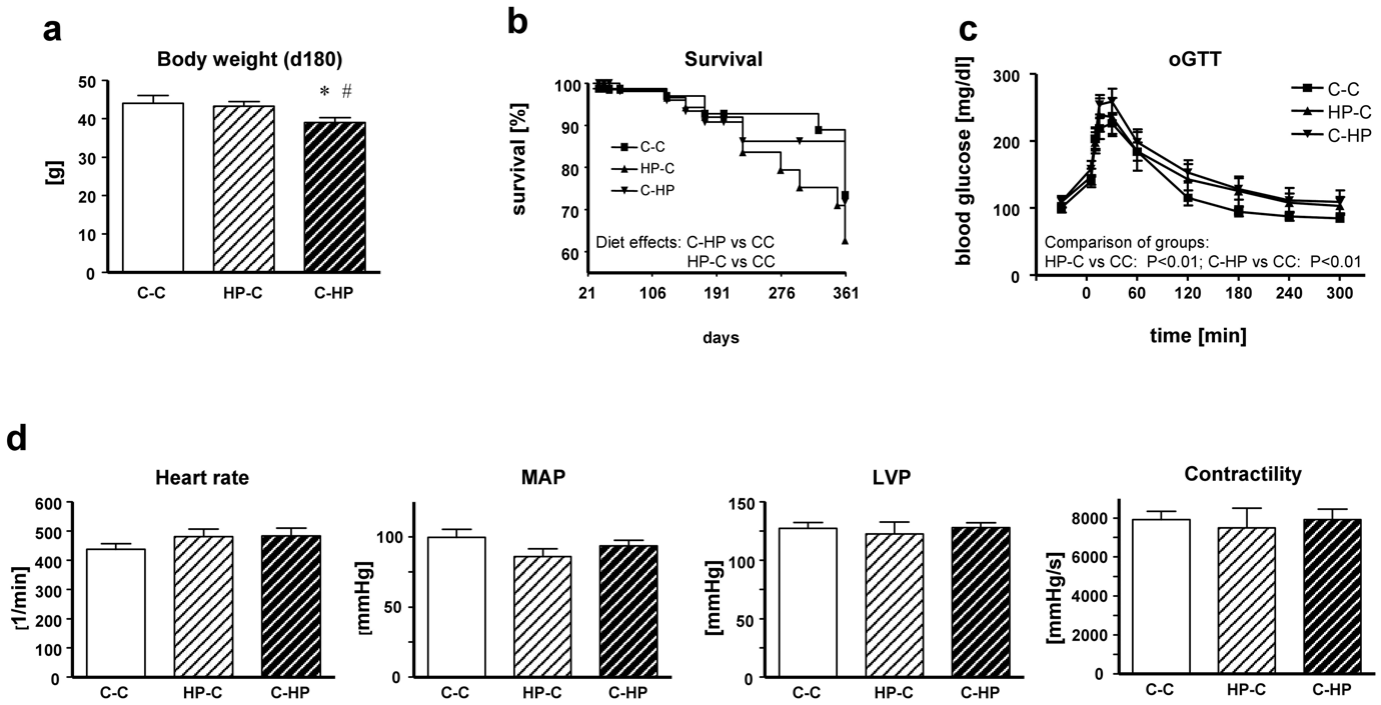
Survival rate and physiological parameters in adulthood. (**a**) Body weight at day 180; (**b**) Oral glucose tolerance test at day 150 (C-C: n = 10; HP-C: n = 7; C-HP: n = 8); (**c**) Kaplan Meier curve from weaning until end of experiment (d360); (**d**) Cardiovascular characterization of mice at day 360 via Tip-catheter (C-C: n = 8; HP-C: n = 7; C-HP: n = 7). Heart rate, left ventricular pressure (LVP), contractility (dP/dtmax), and mean arterial pressure (MAP); Data are the means ± SEM; group differences are calculated by one way of ANOVA (**4a, 4d**) *P<0.05 *vs* control group (C-C); ^#^ *P*<0.05 *vs* high protein-control (HP-C); or two way of ANOVA (**4b**), **P<0.01; group differences (diet effects). For Kaplan-Meier analysis (**4c**) logrank calculation has been performed; ^$^ *P*<0.05.

To check for life-long effects of high-protein diet during pregnancy or lactation, we followed the remaining offspring of all 3 dietary groups (98 C-C; 98 HP-C; 83 C-HP) for one year and measured body weight after 180 and 360 days and biochemical and physiological parameters at the endpoint of the experiment (360 days). The distinctly lower body weight observed in the C-HP group after 21 days of nursing (**Figure 3a**) did not fully normalize after 5 months of normal diet, since the C-HP group at age of 6 months was still characterized by a lower body weight (**Figure 4a**).

To investigate metabolic changes in these animals, we checked alterations in glucose tolerance at an age of 150 days. Fasting blood glucose levels under basal conditions were not significantly altered in either the C-HP or HP-C mice. However, the glucose tolerance test showed that HP exposure during pregnancy or lactation provides an advantage for insulin sensitivity (**Figure 4b**). This agrees with observations that a high-protein diet in adult rats improves glucose tolerance [14]. In contrast, a glucose tolerance test revealed no remaining significant differences between the three treatment groups (at age 300 days, data not shown).

To investigate whether the higher mortality is continued in the C-HP group we calculated survival until the endpoint of our experimental setting (360 days). Kaplan Meier curves revealed no further elevated mortality in the group exposed to maternal HP diet during lactation compared to animals receiving control diet during fetal development and lactation (**Figure 4c**). However, we found a significantly elevated mortality in animals that were born to dams fed a HP diet during their pregnancy, implicating that protein-rich diet during pregnancy might not only have an impact on survival *in utero* and on birth weight, but might influence survival in later life (**Figure 4c**). The reason for the unchanged survival in the C-HP group might relate to a balance between the negative effects of high-protein content during lactation and the beneficial effect of a lower body weight in adulthood [15], [16], [17].

We also characterized cardiovascular parameters at the endpoint of the experimental settings. We found no significant differences for all measured parameters, as exemplarily shown for heart rate, contractility, left ventricular pressure (LVP), or mean arterial pressure (MAP) in **Figure 4d**.

**Figure 5:**
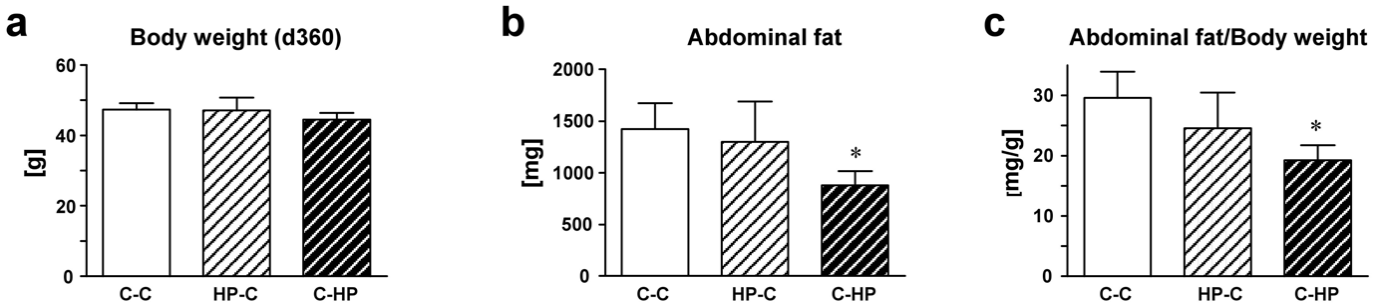
Body weight and fat content at day 360. At the end of the experiment, the following parameters have been determined (C-C: n = 8; HP-C: n = 8; C-HP: n = 8): (**a**) Body weight at day 360; (**b**) Abdominal fat in mice; and (**c**) Abdominal fat/body weight ratio at day 360. Data are the means ± SEM, *P<0.05, *vs* control group (C-C).

We also checked whether the remaining difference in body weight in the C-HP group after 180 days is still existent in an age of 1 year. While there was only a tendency to lower body weight (**Figure 5a**), the measurement of abdominal fat at 360 days showed significantly less fat accumulation in those animals that were exposed to maternal HP diet during lactation (**Figure 5b and 5c**). Thus, following the body weight development until one year of age (d21, d180, d360), the reduced body mass in the C-HP group was highly significant (*P*<0.0001) whereby the difference became less prominent with aging.

Our data demonstrates that a high-protein diet during pregnancy or lactation has distinct and highly relevant effects. While the high protein during pregnancy may particularly affect embryonic lethality, birth weight, and survival in the second half of life, high-protein diet fed to the lactating mother might also have drastic direct effects on offspring during lactation. These direct effects might be responsible for a higher newborn mortality during lactation. Although our findings on diet effects cannot explain exclusively SIDS, because there is a significant amount of literature indicating higher rates of SIDS for mothers who are not breast feeding [18], [19], [20]. Nevertheless, there is broad agreement that the causes for SIDS might be diverse. Therefore, our data offer a first trustable approach to explain the occurrence of SIDS in the group of nursed newborns. Interestingly, an intense debate about relations between maternal nutrition and the occurrence of SIDS has been recently started [21], [22], [23]. Therefore, our findings could be a new and unique link between nutrition and the still incompletely understood human SIDS. Consequently, although offspring of lactating mothers on high-protein diet might have the advantage of lower abdominal fat within the second half of life, this benefit seems not to compensate the immense risk of an early sudden death during lactation.

Taken together our data **may** implicate that both pregnant women and lactating mothers should not follow classical high-protein diets as Atkin's diet.
